# A graph-theoretic criterion for absolute irreducibility of integer-valued polynomials with square-free denominator

**DOI:** 10.1080/00927872.2020.1744618

**Published:** 2020-04-03

**Authors:** Sophie Frisch, Sarah Nakato

**Affiliations:** Institut für Analysis und Zahlentheorie Graz University of Technology, Graz, Austria

**Keywords:** Factorization, non-unique factorization, irreducible elements, absolutely irreducible elements, atoms, strong atoms, atomic domains, integer-valued polynomials, simple graphs, connected graphs, 13A05, 13B25, 13F20, 11R09, 11C08, 13P05

## Abstract

An irreducible element of a commutative ring is absolutely irreducible if no power of it has more than one (essentially different) factorization into irreducibles. In the case of the ring Int(D)={f∈K[x]|f(D)⊆D}, of integer-valued polynomials on a principal ideal domain *D* with quotient field *K*, we give an easy to verify graph-theoretic sufficient condition for an element to be absolutely irreducible and show a partial converse: the condition is necessary and sufficient for polynomials with square-free denominator.

## 1. Introduction

An intriguing feature of non-unique factorization (of elements of an integral domain into irreducibles) is the existence of non-absolutely irreducible elements, that is, irreducible elements some of whose powers allow several essentially different factorizations into irreducibles [1, 6–8, 10].

For rings of integers in number fields, their existence actually characterizes non-unique factorization, as Chapman and Krause [4] have shown.

Here, we investigate absolutely and non-absolutely irreducible elements in the context of non-unique factorization into irreducibles in the ring of integer-valued polynomials on *D*

Int(D)={f∈K[x]|f(D)⊆D},

where *D* is a principal ideal domain and *K* its quotient field.

In an earlier paper [5, Remark 3.9] we already hinted at a graph-theoretic sufficient condition for f∈Int(D) to be irreducible. We spell this out more fully in Theorem 1. This condition is not, however, necessary.

We formulate a similar graph-theoretic sufficient condition for f∈Int(D) to be absolutely irreducible in Theorem 2, and show a partial converse. Namely, our criterion for absolute irreducibility is necessary and sufficient in the special case of polynomials with square-free denominator, cf. Theorem 3.

First, we recall some terminology. Let *R* be a commutative ring with identity.

- r∈R is called *irreducible* in *R* (or, an *atom* of *R*) if it is a non-zero non-unit that is not a product of two non-units of *R*.
- A *factorization* (into irreducibles) of *r* in *R* is an expression
  (1) $$r=a_{1}\cdots a_{n}$$

where n≥1 and *a_i_* is irreducible in *R* for 1≤i≤n.

- r,s∈R are *associated* in *R* if there exists a unit u∈R such that *r* = *us*. We denote this by r∼s.
- Two factorizations into irreducibles of the same element,
  (2) $$r=a_{1}\cdots a_{n}=b_{1}\cdots b_{m},$$

are called *essentially the same* if *n*** **=** ***m* and, after a suitable re-indexing, $a_{j}∼b_{j}$ for 1≤j≤m. Otherwise, the factorizations in (2) are called *essentially different*.

Definition 1.1. Let *R* be a commutative ring with identity. An irreducible element c∈R is called *absolutely irreducible* (or, a *strong atom*), if for all natural numbers *n*, every factorization of *c^n^* is essentially the same as $c^{n}=c\cdots c.$

Note the following fine distinction: an element of *R* that is called *“not absolutely irreducible”* might not be irreducible at all, whereas a *“non-absolutely irreducible”* element is assumed to be irreducible, but not absolutely irreducible.

We now concentrate on integer-valued polynomials over a principal ideal domain.

Recall that a polynomial in D[x], where *D* is a principal ideal domain, is called *primitive* if the greatest common divisor of its coefficients is 1.

Definition 1.2. Let *D* be a principal ideal domain with quotient field *K*, and f∈K[x] a non-zero polynomial. We write *f* as

$$f=\frac{a\prod_{i\in I}g_{i}}{b},$$

where a,b∈D∖{0} with gcd(a,b)=1, *I* a finite (possibly empty) set, and each *g_i_* primitive and irreducible in D[x] and call this the *standard form* of *f*.

We refer to *b* as the *denominator*, to *a* as the *constant factor*, and to $a\prod_{i\in I}g_{i}$ as the *numerator* of *f*, keeping in mind that each of them is well-defined and unique only up to multiplication by units of *D*.

Definition 1.3. For f∈Int(D), the *fixed divisor* of *f*, denoted d(f), is the ideal of *D* generated by *f*(*D*).

An integer-valued polynomial f∈Int(D) with d(f)=D is called *image-primitive*.

When *D* is a principal ideal domain, we may, by abuse of notation, write the generator for the ideal, as in d(f)=c meaning d(f)=cD.

Remark 1.4. Let *D* be a principal ideal domain with quotient field *K*, and f∈K[x] written in standard form as in Definition 1.2. Then *f* is in Int(D) if and only if *b* divides $d(\prod_{i\in I}g_{i}).$

Remark 1.5. Let *D* be a principal ideal domain with quotient field *K*. Then any non-constant irreducible element of Int(D) is necessarily image-primitive. Otherwise, if a prime element p∈D divides d(f), then

$$f=p\cdot \frac{f}{p}$$

is a non-trivial factorization of *f*.

Furthermore, f∈K[x]∖{0} (written in standard form as in Definition 1.2) is an image-primitive element of Int(D) if and only if (up to multiplication by units) *a*** **=** **1 and $b=d(\prod_{i\in I}g_{i}).$

Definition 1.6. Let *D* be a principal ideal domain. For f∈Int(D), and *p* a prime element in *D*, we let

$$d_{p}(f)=v_{p}(d(f))$$

Remark 1.7. By the above definition,

$$d(f)=\prod_{p\in P}p^{d_{p}(f)}\;\text{and}\;d_{p}(f)=\underset{c\in D}{\min}v_{p}(f(c))$$

where P is a set of representatives of the prime elements of *D* up to multiplication by units.

By the nature of the minimum function, the fixed divisor is not multiplicative:

$$d_{p}(f)+d_{p}(g)\leq d_{p}(fg),$$

but the inequality may be strict. Accordingly,

d(f)d(g)|d(fg),

but the division may be strict. Note, however, that

$$d(f^{n})=d{\left(f\right)}^{n}$$

for all f∈Int(D) and n∈N.

## 2. Graph-theoretic irreducibility criteria

We refer to, for instance, [2] for the graph theory terms we use in this section.

Definition 2.1. Let *D* be a principal ideal domain, I≠∅ a finite set and for i∈I, let $g_{i}\in D[x]$ be non-constant and primitive. Let $g(x)=\prod_{i\in I}g_{i},$ and p∈D a prime.

- We say that *g_i_* is *essential* for *p* among the *g_j_* with j∈I if p|d(g) and there exists a w∈D such that $v_{p}(g_{i}(w))>0$ and $v_{p}(g_{j}(w))=0$ for all j∈I∖{i}. Such a *w* is then called a witness for *g_i_* being essential for *p*.
- We say that *g_i_* is *quintessential* for *p* among the *g_j_* with j∈I if p|d(g) and there exists w∈D such that $v_{p}(g_{i}(w))=v_{p}(d(g))$ and $v_{p}(g_{j}(w))=0$ for all j∈I∖{i}. Such a *w* is called a witness for *g_i_* being quintessential for *p*.

We will omit saying “among the *g_j_* with j∈I” if the indexed set of polynomials is clear from the context.

Remark 2.2. When we consider an indexed set of polynomials *g_i_* with i∈I, we are not, in general, requiring $g_{i}\neq g_{j}$ for i≠j. Note, however, that *g_i_* being essential (among the *g_j_* with j∈I) for some prime element p∈D implies gi∼gj in D[x] for all j∈I∖{i}.

Definition 2.3. Let *D* be a principal ideal domain, I≠∅ a finite set and for each i∈I, $g_{i}\in D[x]$ primitive and irreducible.

- The *essential graph* of the indexed set of polynomials $\left(g_{i}|i\in I\right)$ is the simple undirected graph whose set of vertices is *I*, and in which (*i*, *j*) is an edge if and only if there exists a prime element *p* in *D* such that both *g_i_* and *g_j_* are essential for *p* among the *g_k_* with k∈I.
- The *quintessential graph* of the indexed set of polynomials $\left(g_{i}|i\in I\right)$ is the simple undirected graph whose set of vertices is *I*, and in which (*i*, *j*) is an edge if and only if there exists a prime element *p* in *D* such that both *g_i_* and *g_j_* are quintessential for *p* among the *g_k_* with k∈I.

Example 2.4. Let I={1,2,3,4} and for $i\in I,g_{i}\in Z[x]$ as follows:

$$g_{1}=x^{3}-19,\;g_{2}=x^{2}+9,\;g_{3}=x^{2}+1,\;g_{4}=x-5,$$

and set

$$g=(x^{3}-19)(x^{2}+9)(x^{2}+1)(x-5).$$

A quick check shows that the fixed divisor of *g* is 15.

- Taking *w* = 1, 2, 0, respectively, as witnesses, we see that $g_{2},g_{3},g_{4}$ are quintessential for 5. The polynomial *g*_1_ is not essential for 5 because $v_{5}(g_{1}(a))>0$ only if a∈4+5Z and for such *a*, also $v_{5}(g_{2}(a))>0.$
- Taking *w* = 1, 0, 2 respectively, as witnesses, we see that $g_{1},g_{2},g_{4}$ are essential for 3. Only *g*_4_ is quintessential for 3. The polynomial *g*_3_ is not essential for 3.

**Figure 1.**
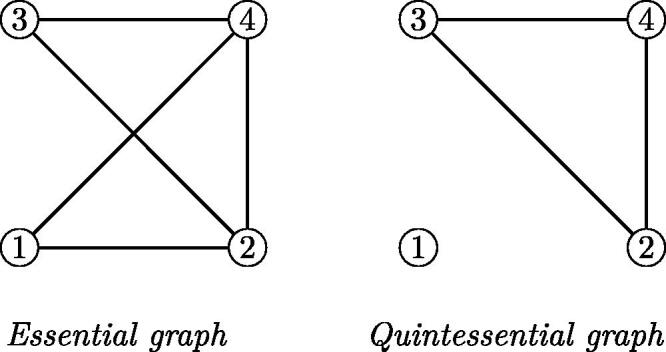
Graphs for example 2.4.

Figure 1 shows the essential and quintessential graphs of $\left(g_{1},g_{2},g_{3},g_{4}\right).$

Lemma 2.5. *Let D be a principal ideal domain and* f∈Int(D) *a non-constant image-primitive integer-valued polynomial, written in standard form according to Definition 1.2 as*

$$f=\frac{\prod_{i\in I}g_{i}}{\prod_{p\in T}p^{e_{p}}},$$

*where T is a finite set of pairwise non-associated primes of D, and let* n∈N.

*Every* h∈Int(D) *dividing f^n^ can be written as*

$$h(x)=\frac{\prod_{i\in I}g_{i}^{\gamma_{i}(h)}}{\prod_{p\in T}p^{\beta_{p}(h)}},$$

*with* $\gamma_{i}(h)\in N_{0}$ *for* i∈I *and unique* $\beta_{p}(h)\in N_{0}$ *for* p∈T*. Moreover, every such representation of h satisfies:*

- *If* q∈T *and* j∈I *such that g_j_ is quintessential for q among the* i∈I*, then*
  $$\beta_{q}(h)=e_{q}\gamma_{j}(h).$$
- *In particular, whenever g_j_ and g_k_ are both quintessential for the same prime* q∈T*, then* $\gamma_{j}(h)=\gamma_{k}(h).$

Proof. We know $d(f^{n})=d{\left(f\right)}^{n}$ (cf. Remark 1.7). So, *f^n^* is image-primitive, and, therefore, all polynomials in Int(D) dividing *f^n^* are image-primitive. Let $f^{n}=hk$ with h,k∈Int(D). When *h* is written in standard form as in Definition 1.2, the fixed divisor of the numerator equals the denominator, and the constant factor is a unit. The same holds for *k*. This is so because *h* and *k* are image-primitive; see Remark 1.5.

Now let q∈D be prime and j∈I such that *g_j_* is quintessential for *q*. Note that, by Remark 2.2 and unique factorization in K[x], the exponent of *g_j_* in the numerator of any factor of *f^n^* is unique.

Writing $f^{n}=hk$ as

$$\frac{\prod_{i\in I}g_{i}^{n}}{\prod_{p\in T}p^{ne_{p}}}=\frac{\prod_{i\in I}g_{i}^{\gamma_{i}(h)}}{\prod_{p\in T}p^{\beta_{p}(h)}}\cdot \frac{\prod_{i\in I}g_{i}^{\gamma_{i}(k)}}{\prod_{p\in T}p^{\beta_{p}(k)}},$$

we observe the following equalities and inequalities of the exponents:

- $ne_{q}=\beta_{q}(h)+\beta_{q}(k)$
- $n=\gamma_{j}(h)+\gamma_{j}(k)$ and hence $ne_{q}=e_{q}\gamma_{j}(h)+e_{q}\gamma_{j}(k)$
- $e_{q}\gamma_{j}(h)\geq \beta_{q}(h)$ and $e_{q}\gamma_{j}(k)\geq \beta_{q}(k).$

(i) follows from unique factorization in *D*.

(ii) follows from unique factorization in K[x] and Remark 2.2.

To see (iii), consider a witness *w* for *g_j_* being quintessential for *q*. Since *f* is image-primitive, $e_{q}=v_{q}(d(\prod_{i\in I}g_{i})),$ by Remark 1.5. From Definition 2.1 and Remark 1.4 we deduce

$$e_{q}\gamma_{j}(h)=v_{q}(g_{j}(w))\gamma_{j}(h)=v_{q}(g_{j}^{\gamma_{j}(h)}(w))=v_{q}\left(\prod_{i\in I}g_{i}{\left(w\right)}^{\gamma_{i}(h)}\right)\geq \beta_{q}(h)$$

(and similarly for *k* instead of *h*).

Finally, (i)–(iii) together imply $e_{q}\gamma_{j}(h)=\beta_{q}(h)$ and $e_{q}\gamma_{j}(k)=\beta_{q}(k).$ □

Theorem 1. *Let D be a principal ideal domain with quotient field K. Let* f∈Int(D) *be a non-constant image-primitive integer-valued polynomial, written in standard form as* f=g/b *with* b∈D∖{0}*, and* $g=\prod_{i\in I}g_{i}$*, where each g_i_ is primitive and irreducible in* D[x].

*If the essential graph of* $\left(g_{i}|i\in I\right)$ *is connected, then f is irreducible in* Int(D).

Proof. If |I|=1, then *f* is irreducible in K[x], and, by being image-primitive, also irreducible in Int(D).

Now assume |I|>1, and suppose *f* can be expressed as a product of *m* non-units $f=f_{1}\cdots f_{m}$ in Int(D). Since d(f)=1, we see immediately that no *f_i_* is a constant, and that $d(f_{k})=1$ for every 1≤k≤m.

Write $f_{k}=h_{k}/b_{k}$ with $b_{k}\in D$ and *h_k_* primitive in D[x]. Then $b=b_{1}\cdots b_{m}$ and there exists a partition of *I* into non-empty pairwise disjoint subsets $I=\cup_{i=1}^{m}I_{k},$ such that $h_{k}=\prod_{i\in I_{k}}g_{i}.$

Select $i\in I_{1}$ and j∈I with j≠i. We show that also $j\in I_{1}.$ Let $i=i_{0},i_{1},\ldots ,i_{s}=j$ be a path from *i* to *j* in the essential graph of $\left(g_{i}|i\in I\right).$ For some prime element *p*_0_ in *D* dividing *b*, $g_{i_{0}}$ and $g_{i_{1}}$ are both essential for *p*_0_. As *g_i_* is essential for *p*_0_, *p*_0_ cannot divide any *b_k_* with k≠1 and, hence, *p*_0_ divides *b*_1_. For any *g_k_* essential for *p*_0_ it follows that $k\in I_{1},$ and, in particular, $i_{1}\in I_{1}.$ The same argument with reference to a prime *p_k_* for which both $g_{i_{k}}$ and $g_{i_{k+1}}$ are essential, shows for any two adjacent vertices $i_{k}$ and $i_{k+1}$ in the path that they pertain to the same *I_k_*, and, finally, that $j\in I_{1}.$

As j∈I was arbitrary, $I_{1}=I$ and *m*** **=** **1. □

Theorem 2. *Let D be a principal ideal domain and* f∈Int(D) *be non-constant and image-primitive, written in standard form as*

$$f=\frac{\prod_{i\in I}g_{i}}{\prod_{p\in T}p^{e_{p}}},$$

*where* I≠∅ *is a finite set and for* i∈I, $g_{i}\in D[x]$ *is primitive and irreducible in* D[x].

*If the quintessential graph G of* $\left(g_{i}|i\in I\right)$ *is connected, then f is absolutely irreducible.*

Proof. Suppose

$$f^{n}=\prod_{l=1}^{s}f_{l},\;\text{where}\;f_{l}=\frac{\prod_{i\in I}g_{i}^{m_{l}(i)}}{\prod_{p\in T}p^{k_{l}(p)}}$$

and $0\leq m_{l}(i)\leq n,0\leq k_{l}(p)\leq ne_{p}$ and for all *i*, $\sum_{l=1}^{s}m_{l}(i)=n$ and for all *p*, $\sum_{l=1}^{s}k_{l}(p)=ne_{p}.$

Fix *t* with 1≤t≤s. We show that *f_t_* is a power of *f* by showing that each *g_i_* with i∈I occurs in the numerator of *f_t_* with the same exponent.

Let i,j∈I. By the connectedness of the quintessential graph, there exists a sequence of indices in *I*, $i=i_{0},i_{1},i_{2},\ldots ,i_{k}=j$ and for each *h*, a prime element *p_h_* in *T* such that $g_{i_{h}}$ and $g_{i_{h+1}}$ are both quintessential for *p_h_*. By Lemma 2.5, $g_{i_{h}}$ and $g_{i_{h+1}}$ occur in the numerator of *f_t_* with the same exponent. Eventually, *g_i_* and *g_j_* occur in the numerator of *f_t_* with the same exponent, for arbitrary i,j∈I. In an image-primitive polynomial, the numerator determines its denominator (as in Remark 1.5) and, hence, *f_t_* is a power of *f*. Since *f_t_* is irreducible, *f_t_* = *f*. □

Example 2.6. The binomial polynomial

$$\left(\begin{array}{c} x\\ p \end{array}\right)=\frac{x(x-1)\cdots (x-p+1)}{p!}$$

where p∈Z is a prime, is absolutely irreducible in Int(Z), by Theorem 2.

The converse of Theorem 2 does not hold in general. For instance, the polynomial

$$f=\frac{x^{2}(x^{2}+3)}{4}\in \text{Int}(Z)$$

is absolutely irreducible in Int(Z) but the quintessential graph of $\left(x,x,x^{2}+3\right)$ is not connected.

There is, however, a converse to Theorem 2 in the special case where the denominator of *f* is square-free, as we now proceed to show, cf. Theorem 3.

## 3. Absolutely irreducible polynomials with square-free denominator

Let *D* be a principal ideal domain with quotient field *K*. When we talk of the denominator of a polynomial in K[x], this refers to the standard form of a polynomial introduced in Definition 1.2.

Remark 3.1. Let *D* be a principal ideal domain. Suppose the denominator of f∈Int(D), written in standard form as in Definition 1.2, is square-free:

$$f=\frac{\prod_{i\in I}g_{i}}{\prod_{p\in T}p}.$$

Then, if *f* is irreducible in Int(D), it follows that each *g_i_* is essential for some p∈T. Otherwise, we can split off *g_i_*. This further implies gi∼gj in D[x] for i≠j, whenever f∈Int(D) with square-free denominator is irreducible. A criterion for irreducibility of an integer-valued polynomial with square-free denominator has been given by Peruginelli [9].

Theorem 3. *Let D be a principal ideal domain and* f∈Int(D) *be non-constant and image-primitive, with square-free denominator, written in standard form as*

$$f=\frac{\prod_{i\in I}g_{i}}{\prod_{p\in T}p},$$

*where* I≠∅ *is a finite set and for* i∈I, $g_{i}\in D[x]$ *is primitive and irreducible in* D[x].

Let G be the quintessential graph of $\left(g_{i}|i\in I\right)$ as in Definition 2.3.

Then f is absolutely irreducible if and only if G is connected.

Proof. In view of Theorem 2, we only need to show necessity. If |I|=1, then *G* is connected. Now assume |I|>1, and suppose *G* is not connected. We show that *f* is not absolutely irreducible. If *f* is not even irreducible, we are done. So suppose *f* is irreducible. This implies gi∼gj in D[x] for i≠j, by Remark 3.1. Since *G* is not connected, *I* is a disjoint union of *J*_1_ and *J*_2_, both non-empty, such that there is no edge (*i*, *j*) with $i\in J_{1}$ and $j\in J_{2}.$

We express *T* as a disjoint union of *T*_1_ and *T*_2_ by assigning every p∈T for which some $g_{i}\in J_{1}$ is quintessential to *T*_1_, every p∈T for which some $g_{i}\in J_{2}$ is quintessential to *T*_2_, and assigning each p∈T for which no *g_i_* is quintessential to *T*_1_ or *T*_2_ arbitrarily. (It may happen that $T_{1}=\emptyset$ and $T_{2}=T$ or vice versa).

Then *f* ^3^ factors in Int(D) as follows:

$$f^{3}=\frac{{\left(\prod_{i\in J_{1}}g_{i}\right)}^{2}\prod_{j\in J_{2}}g_{j}}{{\left(\prod_{p\in T_{1}}p\right)}^{2}\prod_{q\in T_{2}}q}\cdot \frac{{\left(\prod_{j\in J_{2}}g_{j}\right)}^{2}\prod_{i\in J_{1}}g_{i}}{{\left(\prod_{q\in T_{2}}q\right)}^{2}\prod_{p\in T_{1}}p}.$$

As Int(D) is atomic (cf. [3]), each of the two factors above can further be factored into irreducibles. Since *J*_1_ and *J*_2_ are both non-empty and gi∼gj in D[x] (and hence, gi∼gj in K[x]) for i≠j, it is clear that the resulting factorization of *f*^3^ into irreducibles is essentially different from f·f·f. □

Remark 3.2. Let *D* be a principal ideal domain. The proof of Theorem 3 shows that any non-absolutely irreducible element f∈Int(D) with square-free denominator exhibits non-unique factorization of *f^n^* already for *n*** **=** **3.

If $f(x)=\prod_{i\in I}g_{i}(x)/p,$ where *D* is a principal ideal domain, *p* a prime of *D* and each $g_{i}\in D[x]$ primitive and irreducible in D[x], then it is easy to see that *f* is an irreducible element of Int(D) if and only if

- $d(\prod_{i\in I}g_{i}(x))=p$ and
- each *g_i_* is essential for *p*, that is, for each i∈I there exists $w_{i}\in D$ such that $v_{p}(g_{i}(w_{i}))>0$ and $v_{p}(g_{j}(w_{i}))=0$ for all j∈I∖{i}.

An analogous statement relates absolutely irreducible integer-valued polynomials with prime denominator to quintessential irreducible factors of the numerator:

Corollary 3.3. *Let D be a principal ideal domain,* p∈D *a prime, and* I≠∅ *a finite set. For* i∈I*, let* $g_{i}\in D[x]$ *be primitive and irreducible in* D[x]*. Let*

$$f(x)=\frac{\prod_{i\in I}g_{i}(x)}{p}.$$

*Then f is an absolutely irreducible element of* Int(D) if and only if

- $d(\prod_{i\in I}g_{i}(x))=p$ *and*
- each *g_i_ is quintessential for p among the g_i_ with* i∈I*, that is, for each* i∈I *there exists* $w_{i}\in D$ *such that* $v_{p}(g_{i}(w_{i}))=1$ *and* $v_{p}(g_{j}(w_{i}))=0$ *for all* j∈I∖{i}.

Proof. If $d(\prod_{i\in I}g_{i}(x))=p,$ then f∈Int(D) with d(f)=1, and Theorem 3 applies. If, on the other hand, *f* is in Int(D) and is absolutely irreducible, then *f* is, in particular, irreducible and therefore d(f)=1, and, again, Theorem 3 applies. Now the statement follows from the fact that, whenever $d(\prod_{i\in I}g_{i}(x))=p$ is prime, the quintessential graph of $\left(g_{i}|i\in I\right)$ is connected if and only if every *g_i_* is quintessential for *p*. □

We conclude by an example of how to apply Theorem 3:

Example 3.4. The following polynomial f∈Int(Z) is irreducible, by Theorem 1; but not absolutely irreducible, by Theorem 3:

$$f=\frac{\left(x^{3}-19)(x^{2}+9)(x^{2}+1)(x-5\right)}{15}$$

This is so because the essential graph of $\left(x^{3}-19,x^{2}+9,x^{2}+1,x-5\right)$ is connected, but the quintessential graph is not connected, see Example 2.4 and Figure 1.
